# The role of diabetes distress in Diabulimia

**DOI:** 10.1186/s40337-023-00924-7

**Published:** 2023-12-01

**Authors:** Stephen Poos, Misha Faerovitch, Celeste Pinto, Nima Jamalkhani, Fahad Chaudhri, Satara Khan, David F. Lo, Kaitlin McGowan, Ashaki Martin

**Affiliations:** 1 Stony Brook Southampton Hospital, 240 Meeting House Ln, Southampton, NY USA; 2 School of Medicine and Dentistry, University of Rochester, 601 Elmwood Ave, Rochester, NY USA; 3 Rowan-Virtua School of Osteopathic Medicine, 1 Medical Center Dr, Stratford, NJ USA; 4 Touro University, 3 Times Sq, New York, NY USA; 5 Dartmouth Hitchcock Medical Center, 1 Medical Center Dr, Lebanon, NH USA; 6 Ocean University Medical Center, 425 Jack Martin Blvd, Brick, NJ USA

**Keywords:** Diabetes distress, Diabulimia, Eating disorders, Type 1 diabetes

## Abstract

**Importance:**

Diabulimia is a disordered eating behavior in which a person with type 1 diabetes withholds insulin injections to lose weight. It is thought that the psychosocial stress of managing this chronic disease, which is termed diabetes distress, may contribute to developing diabulimia.

**Objective:**

This paper explores links between diabetes distress and diabulimia and their relevance to the diagnosis and treatment of diabulimia by assessing whether people with diabulimia report measurable evidence of diabetes distress. Evidence Review: We evaluated studies examining the qualitative experiences of people with disordered eating behaviors in the setting of type 1 diabetes for themes of diabetes distress by identifying aspects of the patients’ stories that matched the criteria in the Diabetes Distress Scale. Selected studies recorded primary data, analyzed qualitative data, examined lived experiences of individuals with diabulimia, and were made available in English-language peer-reviewed journals between January 1, 2000 and August 31, 2022. Exclusion criteria included partial articles, editorials, reviews, and abstracts along with studies of patients with type 2 diabetes.

Findings.

Over forty individual participants across twelve studies were found to have aspects of their experiences that met one or more criteria from the Diabetes Distress Scale. Participants reported experiences that matched criteria items from each of the seven subscales of the Diabetes Distress Scale. Participants in the twelve studies included 185 individuals with type 1 diabetes experiencing diabulimia, including 164 females (88.6%), 20 males (10.8%), and 1 non-reported gender (0.54%).

**Conclusion:**

We believe this discovery warrants further research probing the prevalence of diabetes distress among people with diabulimia as well as other links between the two conditions. We advocate for a diabetes distress-informed approach to diabulimia treatment and for diabetes distress screening in every patient with type 1 diabetes.

## Highlights

- It is thought that the psychosocial stress of managing diabetes, termed diabetes distress, may contribute to developing diabulimia, which is a disordered eating behavior in which a person withholds insulin injections to lose weight.
- We explore links between diabetes distress and diabulimia and their relevance to the diagnosis and treatment of diabulimia.
- Studies were evaluated by examining the qualitative experiences of people with disordered eating behaviors in the setting of type 1 diabetes for themes of diabetes distress by identifying aspects of the patients’ stories that matched the criteria in the Diabetes Distress Scale.
- Over forty individual participants across twelve studies were found to have aspects of their experiences that met one or more criteria from the Diabetes Distress Scale.
- We believe this discovery warrants further research probing the prevalence of diabetes distress among people with diabulimia as well as other links between the two conditions.

## Abstract (plain language summary)

Diabulimia is a disordered eating behavior in people with type 1 diabetes who intentionally withhold insulin injections to lose weight. This study investigates the connection between diabulimia and diabetes distress, the daily psychosocial stress of managing diabetes. We examined the qualitative narratives of 40 participants with diabulimia as recorded in 12 studies and found that they reported experiences matching criteria from the Diabetes Distress Scale, a tool to measure diabetes-related stress. We suggest further research to understand the prevalence of diabetes distress in people with diabulimia. Also, we advocate for using a diabetes distress-informed approach in diabulimia treatment, including screening for diabetes distress in all individuals with type 1 diabetes.

## Background

Diabulimia describes the behavior of patients with type 1 diabetes (T1D) who deliberately administer inadequate insulin to themselves for the purpose of weight loss [1]. Exogenous insulin administration, the mainstay of T1D management, frequently leads to weight gain [2]; withholding insulin may mitigate this side effect, but with deleterious consequences. Recurrent diabetic ketoacidosis (DKA) and poor glycemic control, hallmarks of insulin restriction [3], and result in increased rates of complications such as neuropathy, retinopathy, and hospitalization [4]. However, many individuals with diabulimia often persist with the behavior despite full awareness of the potential detrimental medical consequences [5]. Diabulimia does not appear as a distinct diagnosis in the DSM-V but is categorized as a disordered eating behavior, a broad term encompassing behaviors that do not meet the criteria of a specific DSM-defined eating disorder [6]. However, researchers are beginning to appreciate its prevalence in the population of patients with diabetes, especially among patients identifying as female. In a United States study conducted in females with T1D ranging in age from 13 to 60 years, 31% reported having intentionally omitted insulin over the course of their disease, with nearly 9% of respondents indicating that this behavior occurred frequently. Among those omitting insulin, half stated that weight control was the primary reason for their behavior [4].

Since the 1990s, researchers have probed the psychological effects of diabetes self-management on patients. Termed diabetes distress (DD), it affects between 20 and 40 percent of patients with T1D [7]. This stress may be comorbid with a depressive disorder, but is distinct from it [7]. DD may be measured using the Problem Areas in Diabetes (PAID) survey or the Diabetes Distress Scale (DDS), whose subscales allow for evaluation of different facets of DD. Elevated scores on the PAID, DDS, and the regimen-related distress subscale of the DDS are correlated with poorer glycemic control in patients with T1D [8]. The combination of the tendency of patients with diabetes distress to exhibit lower levels of self-care with the physiological and emotional components of stress is thought to be the mechanism behind the consistently observable outcome of diabulimia [7].

Several factors contribute to the development of DD. Many individuals with diabetes, especially type 1, are concerned about weight gain as insulin therapy can lead to increased fat storage. This can lead to body image issues due to concerns about insulin-induced weight gain or disruptions to their physical appearance [9]. The stigma and misunderstanding surrounding diabetes can lead to feelings of isolation and social pressure to maintain a certain body image [10]. This exaggerated self-awareness is heightened by the emphasis on meticulous diet and medication regimens from health care providers and family members [9]. The constant management of diabetes can lead to significant psychological distress, including anxiety, depression, and burnout [9]. Patients report fear of diabetes-related health complications and feel overwhelmed by the management of their condition. Desire for control over their lives may contribute to disordered eating behaviors such as diabulimia [10].

Research regarding the experiences of people with diabulimia has revealed their detrimental experiences with T1D and their resulting distress [10, 11]. This paper explores the way diabetes distress impacts diabulimia patients and its relevance to the diagnosis and treatment of diabulimia.

## Methods

Our objective was to establish whether people with T1D with a comorbid eating disorder or disordered eating behaviors such as diabulimia are experiencing signs of diabetes distress. We searched for primary articles that contained complete interviews of people with diabulimia or T1D with a comorbid eating disorder. We also included Goddard and Oxlad’s 2022 meta-synthesis of twelve studies containing primary qualitative patient data within articles that broadly discussed insulin omission or restriction in T1D and eating disorders or disordered eating behaviors in T1D [10].

### Search strategy

**Fig. 1:**
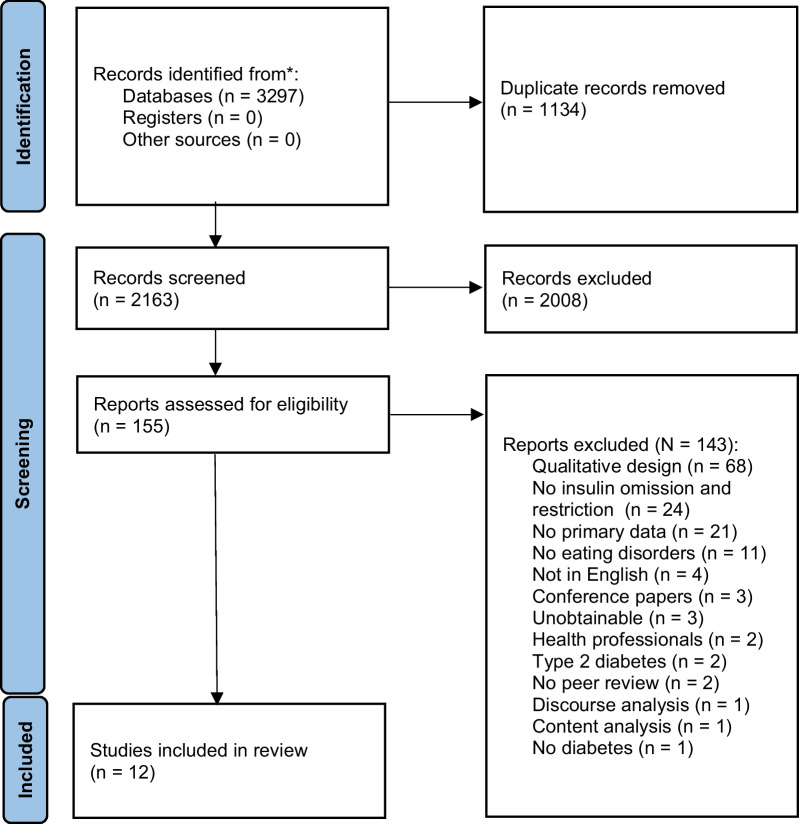
*PRISMA* flow diagram [12] demonstrating the article selection process adapted from Goddard and Oxlad, 2022

In accordance with Preferred Reporting Items for Systematic Reviews and Meta-analyses (PRISMA) guidelines, six electronic databases—CINAHL, Embase, PsychINFO, PubMed, Scopus, and Web of Science—were systematically searched for studies published between January 1, 2000 and August 31, 2022. Search terms included MeSH terms ((“Role” OR “Function” OR “Criteria”) AND (“Diabetes” OR “High Blood Sugar” OR High Blood Glucose”) AND (“Distress” OR “Psychological Effects”) AND (“Diabulimia” OR “Eating Disorders” OR “Insulin Omission”)). Cited references of included studies were also evaluated for inclusion (Fig. 1).

All retrieved studies were initially screened according to inclusion criteria through review of titles and abstracts. If study design was unclear from title or abstract review, the full text was reviewed for eligibility. Selected studies recorded primary data, analyzed qualitative data, examined lived experiences of individuals with diabulimia, and were available in peer-reviewed journals in English. Data was classified as qualitative if it was obtained or analyzed using qualitative methods. Data on insulin restriction/omission for weight control was also included if it was extractable and reported separately from data regarding eating disorders and behaviors broadly in type 1 diabetes patients.

Exclusion criteria included partial articles, editorials, reviews, and abstracts along with studies of people type 2 diabetes. Interventions consisting solely of a brief follow-up contact were also excluded, as these have been reviewed previously.

### Data abstraction, evaluation, and synthesis

All studies were scored using the QualSyst Quality Assessment Checklist (Kmet et al., 2004), examining methodological rigor in ten central elements of study validity. These scores (‘yes’ 2, ‘partial’ 1, ‘no’ 0) were tallied and then divided by 20 (total possible score), resulting in scores between 0 and 1. All twelve papers had scores above 0.65, representing better quality studies. Relevant data that met the following criteria was abstracted: study setting, sample characteristics, diabulimia experiences, study attributes, and eating disorder characteristics. Study design, risk of bias, and threats to inference in each study was not assessed.

While this meta-synthesis analyzed patient experiences and provided evidence-based guidance for practice, it did not specifically evaluate individual DD symptoms in the patients that are the focus of the present study. These twelve articles contained verbatim transcriptions of patient responses and/or qualitative thematic analysis. These responses/analyses were reviewed to identify aspects that matched the criteria in the 28-item T1D Diabetes Distress Scale (c), which was chosen for its ability to evaluate seven subscales. The questionnaire can be administered and completed in approximately five to ten minutes with the seven subscales of the T1D-DDS and their definitions are reproduced below [13]:

1. Powerlessness: A broad sense of feeling discouraged about diabetes
2. Management Distress: Disappointment with your self-care efforts
3. Hypoglycemia Distress: Concerns about hypoglycemic events
4. Negative Social Perception Distress: Concerns about the possible negative judgments of others
5. Eating Distress: Concerns that your eating is out of control
6. Physician Distress: Disappointment with your current health care professionals
7. Friend/Family Distress: Perception of too much focus on diabetes amongst your loved ones

The reported symptoms and experiences of the participants in each of the cited studies were evaluated using the T1D-DDS. To ascertain reliability, several measures were implemented. Firstly, we maintained methodological consistency by using standardized procedures and consistent methodologies for data collection and analysis, which minimized the potential for variations in the results arising from differences in data collection or analysis techniques. All authors were instructed to interpret the criteria items similarly to ensure inter-rater agreement. We subjected the results to peer review, group discussion and validation by all the authors on the paper. These comprehensive measures were undertaken to ensure the reliability of the evaluation of T1D-DDS criteria items, making the findings dependable and suitable as a foundation for informed decisions in the context of diabetes distress assessment and management.

## Results

Of the twelve studies that met the inclusion criteria, there was one study each from Brazil, America, Australia, Portugal, and Ireland with the remaining seven coming from the United Kingdom. The means of data collection consisted of online blogs (N = 1), online focus groups (N = 1), physical focus groups (N = 2), self-reported questionnaires (N = 2), and interviews (N = 6). Within the twelve studies, the various techniques used to analyze data included employed content analysis (N = 2), phenomenological analysis (N = 1), discourse analysis (N = 1) and constructivist grounded theory approach (N = 1). The remainder (N = 7) utilize thematic analysis, (Table 1).

**Table 1:** Summary of studies (N = 12)

Author (Country)	Year	Methodology				
		Recruitment	Collection	Sample size (*n =* 195)	Analysis	Quality score
Balfe [14] (Ireland)	2013	Diabetes centers, Diabetes Ireland Facebook page	Interview	6	Thematic	0.85
Coleman [5] (UK)	2020	Diabetics with Eating Disorders and Diabetes Daily; Private Facebook group ‘Diabulimia Awareness’	16 open-ended questions, online	45	Thematic	0.80
Falcao [15] (Portugal)	2017	Diabetes social networks	20-item self-report survey	55	Content	0.85
Harrison [16] (UK)	2021	King’s Health Partners, Royal Free & Royal Bournemouth Hospitals; Social media	2 semi-structured interviews	13	Construct-ivist	0.90
Hastings [17] (UK)	2016	‘Diabulimia’ online group	Focus group online	13	Thematic	0.90
Hillege [18] (Australia)	2008	Self-help/support groups (The Juvenile Diabetes Foundation and Diabetes Australia)	Semi-structured interviews	4	Content	0.70
Kay [13] (UK)	2009	City-wide diabetes service	Semi-structured interviews	9	Phenomen-ological	1.00
Macdonald [19] (UK)	2018	National Charity (Diabetes with Eating Disorders); Professional networking	Semi-structured interviews	9	Thematic	0.90
Powers [20] (USA)	2016	Park Nicollet Health Services (PNHS)	Focus group	16	Thematic	0.90
Ribeiro [21] (Brazil)	2021	Facebook group ‘Diabetes E Diabulimia’	Interviews	4	Discourse	0.65
Staite [9] (UK)	2018	Blogs found on Google	Online Blogs	11	Thematic	0.95
Wilson [11] (UK)	2012	Diabetes meeting following the Insulin Pump Therapy (INPUT) group	Focus Group	10	Thematic	0.65

Participants in the twelve studies included 185 individuals with T1D experiencing diabulimia, including 164 female (88.6%), 20 male (10.8%), and 1 non-reported gender (0.54%). Unfortunately, not all studies included demographic and ethnicity information. The only ethnicities reported were Caucasian (n = 50, 27%) and Brazilian (n = 4, 2.2%). The rest were either not reported (n = 125, 67.6%) or reported as unknown (n = 6, 3.2%). Nine studies reported age, with a total of 121 participants aged 15–67 years and an average age of 28.1 years (SD = 8.84).

Analyzing research that explores the experience of T1D patients with diabulimia and other eating disorders displays themes that correlate to the themes featured in T1-DDS, a scale used to measure distress as it relates to managing diabetes. The DDS features seven subscales: Powerlessness (PL), Management Distress (MD), Hypoglycemia Distress (HD), Negative Social Perceptions (NSD), Eating Distress (ED), Physician Distress (PD), and Family/Friend Distress (FFD). Themes in the primary sources that matched item criteria under each subscale are discussed below and summarized in Table 2 with numbers indicating the criteria items from each subscale that the participant meets.

**Table 2:** DDS criteria items met by participants' experiences (N = 45)

Participant	Study Author	DDS Subscales						
		PL	MD	HD	NSP	ED	PD	FFD
Ps 23	Balfe							20
Ps 24	Balfe					23		
24-year-old	Falcao	25						
Ps 9	Hastings							11, 17, 20
Ps 10	Hastings						14, 26	
Ps 12	Hastings			3				
Ps 81	Hastings				4			
Ps 93	Hastings						26	
Ps 2	Hillege						7, 14	
Ps 3	Hillege						18	
Ps 4	Hillege		1			23		
Ps 5	Hillege	25						
Ps 6	Hillege	25	1		24			
Ps 7	Hillege							
Ps 8	Hillege				10			
Ps 9	Hillege				4, 24			
Ps 10	Hillege							6
Ps 11	Hillege							20
Ps 12	Hillege							11, 20
Charlotte	Kay				4			
Francis	Kay	25			4, 19, 24			
Jo	Kay		1	27		16		6
Joanne	Kay	21, 25						
Lisa	Kay					16	18	
Melanie	Kay				24	16		20
Rachel	Kay		1					20
Samantha	Kay	25	1		4, 19, 24	2		
Sophie	Kay	25	1, 28		24			
Ps 1	MacDonald					16	26	
Ps 3	MacDonald				4		18	
Ps 6	MacDonald					23		
Ps 7	MacDonald					23		
Ps 8	MacDonald							6
Narrator 3	Ribeiro		1				7, 14, 26	
Ps 111	Staite				4, 10, 24			
Ps 119b	Staite	13, 21, 25						
33-year-old	Wilson			3				
38-year-old	Wilson		1, 28					
44-year-old	Wilson		1, 12					
44-year-old	Wilson		1, 28					
46-year-old	Wilson	5, 13, 21, 25						
48-year-old	Wilson			3, 27				
Unidentified	Coleman				4, 10			
Unidentified	Harrison	21, 25	8, 12, 28	27	10	23		
Unidentified	Powers	9, 21, 25				16		
Total identified (N)		9	10	4	11	9	9	9
*Total inc. unidentified*		11	11	5	13	10	9	9
Most common criteria		25	1	27	4	23	26	20

Patients from each study meeting at least one DDS criteria item are displayed here, with the number of the criteria item appearing in its subscale column. In nine of the twelve studies, patients are individually identified by their participant or narrator number, age, or first name. The remaining three studies make no such distinctions. At the bottom of the table, a tally for each DDS subscale was made of the number of participants who met at least one criteria item from within that subscale. The numeral of the most commonly met criteria item within each subscale was recorded as well. Abbreviations: Ps, Participant; PL, Powerlessness; MD, Management Distress; HD, Hypoglycemia Distress; NSP, Negative Social Perceptions; ED, Eating Distress; PD, Physician Distress; FFD, Friend/Family Distress

### Powerlessness

The theme of Powerlessness featured in many of the patients’ testimonials about their experiences. Some expressed feelings of discouragement after seeing unexplainable high blood glucose numbers and despair caused by the unpleasant side effects that correspond with those numbers [18]. Others worried extensively about the development of long-term complications despite any effort they put into managing their diabetes, adding more complications to their lives [11]. These patients also expressed feeling that they must perfectly manage their diabetes or risk developing complications in the future [18]. However, the most prevalent aspect of powerlessness that appeared throughout many of the patients’ interviews was the belief that their efforts to manage their diabetes would never be good enough. Many patients cited feeling stuck in a cycle where, as one patient put it, “even when I’m trying to cut down food, my blood will go low or something and then I end up eating again so it’s just defeated the whole object of my day” [16].

### Management distress

The theme of management distress encompasses ideas surrounding patients’ feelings about their skills and abilities to care for their diabetes. An element of this theme seen in a few patient accounts regard patient concerns about not check their blood glucose enough, resulting in not knowing their levels and that they contributed to negative health consequences [18]. Another patient concern pertains to not giving their diabetes enough attention by properly taking their insulin so that they now face significant health issues that affect their daily life [18]. The most prevalent element of this theme was patients’ general feeling that they are not as skilled as they should be to deal with their diabetes. One patient described how their history of negative thinking led to “cutting myself up, cause I hated myself…I didn’t feel like a strong person cause I wasn’t dealing with this and it went on and I wasn’t looking after myself” [16].

### Hypoglycemia distress

The theme of hypoglycemia distress from the T1-DDS scale does not feature heavily in the literature overall. A few patients stated that they have difficulty recognizing the warning signs of hypoglycemia. One patient stated that so many factors that can impact blood glucose levels make it difficult to know the right choice for your health [19]. Other patients describe the anxiety they experience from never feeling safe from the possibility of a serious hypoglycemic event. This anxiety manifests as feeling like “I can’t allow my blood glucose to drop because it makes me feel too anxious about having a hypo” [14]. Despite these accounts, this subscale of the DDS had the fewest matches in the literature.

### Negative social perceptions

The theme of negative social perceptions centers around patients’ feelings about how others may associate negative connotations with their diabetes and change their treatment of patients in social settings. One element of this theme reflected in patient surveys involved patients feeling that they had to hide their diabetes from others. Many patients specifically stated they tried to refrain from injecting insulin in public to avoid judgments from others [5, 14]. Some participants worried that their diabetes would cause peers and employers to think less of them [17]. The most prominent element of this theme in the literature centered around patients feeling that others would treat them differently because of their diabetes. One patient described classmates being jealous because they were allowed to eat in class when necessary and people being judgmental about their weight when eating sugar in public to avoid a hypoglycemic event [13].

### Eating distress

The theme of eating distress centers around patients’ feelings toward food and the sense that their eating habits control their lives. One aspect of this theme that is minimally represented in patient interviews is their feeling that they do not eat as carefully as they should. One patient describes a constant worry about what they are eating and maintaining their healthy lifestyle [16]. A more heavily represented aspect of the eating distress theme in the patients’ accounts from the literature is the feeling that thoughts of food and eating control the patients’ lives. One patient stated that their diabetes caused them to be so focused on food and aware of calories that it worsened their disordered eating behavior [22, 23]. The most prevalent element seen in the patient interviews was patients’ feelings that their eating was out of control. Many patients talked about experiences of eating constantly and adjusting their insulin to compensate for perceived weight gain. One patient recounted that they repeatedly “ate a week's normal person calories in a day” [24] and then would decide not to take insulin for fear of gaining weight.

### Physician distress

The theme of physician distress centers around a perceived disconnect between the patient and their physician regarding the experience of living with diabetes, and some patients cited this as contributing significantly to the distress they felt regarding their diabetes. Some patients reported feeling unable to openly communicate with their physician about their thoughts and feelings related to their illness. One patient describes not wanting to go to the endocrinologist and lying about their blood sugar results for fear of being scolded by the physician [16]. Another aspect of this theme that patients reported was feeling that they did not get the help from their doctor that they needed to manage their diabetes. These patients felt that physicians did not listen to or understand patients’ experiences and circumstances [19]. Patients cite this feeling that their physicians do not understand what it is like to live with diabetes as another source of distress, stating that “you just expect that people won’t get it, so you’ve already got this attitude of they’re not going to understand me” [22, 23]. The most predominant element of this theme that featured in many patient accounts was feeling that their physician did not know enough about diabetes or caring for patients with diabetes, citing incidences where healthcare professionals forgot to do blood tests or give them the insulin that patients with eating disorders already struggle with taking [22, 23].

### Family/friend distress

The theme of family/friend distress encapsulates patients’ feelings that their loved ones added to the burden of their illness. One element of this theme featured in the literature is patients’ feeling that their family and friends enforced their diabetes management more than the patient felt necessary. Some patients stated that their loved ones become anxious or panicked about the patient’s diabetes [22, 23]. Another element of this theme mentioned by a few patients was family and friends worrying about hypoglycemia more than the patient would like. One patient cites their partner repeatedly pushing them to eat to avoid a hypoglycemic event even when the patient felt it was not necessary [16]. The most significant featured element of family/friend distress evident in the literature was feeling as though family members and friends monitored the patient’s diabetes too closely. One patient described how their parents hid food and offered to put a lock on the refrigerator at night, which made the patient “[hate] them because of it and then I’d eat everything” [24].

## Discussion

Nine of the twelve studies utilized unique patient identifiers, which made it possible to view single participants in the studies through the lens of DD. Several individual participants reported experiences matching multiple DDS item criteria across various subscales. While no single participant was reported as experiencing criteria in more than three subscales, it can be inferred that more life experiences meeting DD criteria could have been uncovered if these people had been administered a DDS.

The primary sources reviewed in this study reported the most participant experiences within the Negative Social Experiences subscale, while issues regarding Hypoglycemia Distress received the least attention, far less than any of the others. This could be reflective of patients’ efforts to prioritize outward appearance and weight, and thereby their perceived social status, over their own health and safety. This conclusion appears to be supported by the results of the meta-synthesis by Goddard and Oxlad [13]. Using the DDS, our research was able to elucidate more specific themes within the patients’ experiences. Commonly met DDS item criteria included “Feeling that people treat me differently when they find out I have diabetes” (item 4), “Feeling that my friends or family act like ‘diabetes police’” (item 20), “Feeling that I am not as skilled at managing diabetes as I should be” (item 1), “Feeling that no matter how hard I try with my diabetes, it will never be good enough” (item 25) [13]. These insights begin to build a profile of an archetypal patient whose feelings of low efficacy and perceived inability to live up to the standards of diabetes management cause them to seek validation in other ways, including harmful weight loss methods such as insulin restriction.

This archetypal patient model is echoed by De Paoli and Rogers, who found that the difficulty in T1D management creates frustration among patients, which leads to negative perceptions of themselves that, in turn, can prompt disordered eating behaviors [25]. Goddard and Oxlad view disordered eating behavior as patients’ attempts to regain the control they feel that diabetes has stolen from them [10]. Occasional insulin restriction yields the desired result of weight loss, but positive perceptions of this behavior are replaced with feelings of guilt as it becomes more habitual and self-management is neglected. As insulin restriction escalates, so does the feeling of distress. Patients who go on to experience diabetes-related complications because of their insulin restriction also express guilt over their prior actions [10].

The literature provides a context for this proposed model of DD leading to disordered eating behavior or eating disorders in T1D. The risk of developing an eating disorder or disordered eating behavior continues to rise as children with T1D progress through adolescence and into their twenties [26]. In addition, experiencing symptoms of DD within an hour of eating increases the risk of binge eating behavior [25], which raises the likelihood of DD influencing other eating behaviors. Because of the heightened risk that a person with T1D will develop disordered eating behaviors, such as diabulimia, or eating disorders [9], the distress experienced by these patients must be addressed in their treatment and recovery.

### Implications for management and prevention

Non-compliance with treatment in young people with T1D is reason for initiating screening for an eating disorder or disordered eating behavior, especially in the setting of risk factors such as female gender, high body mass index prior to T1D diagnosis, diagnosis of T1D between 7 and 18 years of age, dissatisfaction with bodily appearance, and low self-esteem [9]. Frequently used screening instruments include the modified Eating Disorder Inventory (mEDI), Diabetes Eating Problem Survey (DEPS), or the modified SCOFF (mSCOFF) questionnaire [26]. The letters in mSCOFF are partly derived from an acronym for its criteria, with a score of two or more suggesting an eating disorder:

1. Do you make yourself Sick because you feel uncomfortably full?
2. Do you worry you have lost Control over how much you eat?
3. Have you recently lost > 14 pounds (One stone) in a 3-month period?
4. Do you believe yourself to be Fat when others say you are too thin?
5. Do you ever take less Insulin than you should?

In light of the results of this study, the clinicians may consider utilizing the PAID scale or DDS to evaluate for the presence of underlying DD as a driver for disordered eating behaviors [10]. Candler, et al. propose a multidisciplinary approach to diabulimia consisting of a diabetes management team, dietician team, and mental health team [26]. Considerations for the diabetes management team informed by the presence of DD include emphasizing “good enough” glycemic control over “optimal” [3]. Decreasing time spent on diabetes self-management can decrease diabetes distress and thus can play a role in diabulimia recovery [3]. While diabetes care providers may be reluctant to relax their treatment regimens, having patients spend less time on their diabetes management is a part of the recovery process as patients work toward more incremental health goals [3, 9]. It is also important to involve the patient in the decisions made for further diagnosis and treatment [27].

For the dietician team, goals should include re-establishing a regular meal pattern, establishing intuitive approaches to meal planning, and potentially relaxing carbohydrate counting [26]. Providers should avoid giving positive reinforcement for any weight loss that occurs during recovery because of the potential for sabotaging patient recovery, especially in the setting of diabulimia [3]. Online group seminars addressing the facts of diabetes diet and management have been found to be as efficacious in reducing DD and hemoglobin A1C as online group meetings discussing the emotional aspects of diabetes management [28]. However, those with poor emotion regulation or poor cognitive skills benefited the most from the emotion-focused intervention [28], with patients reporting long-term gains in emotional perspective about the realities of managing T1D and decreased self-blame [29]. Dieticians will need to be prepared to address the non-productive emotions of their patients with diabulimia and DD and work closely with the mental health team when treating a patient with poor emotion regulation abilities.

The mental health team should conduct cognitive behavioral therapy and assess and address the impacts of the T1D and eating disorder on daily living [9]. DD-informed talk therapy may touch on themes of attachment theory and relationships. A role for attachment theory has been proposed for patients’ ability to self-manage their T1D, as secure attachments in interpersonal relationships are associated with better glycemic control across multiple studies [30]. Underscoring this idea is that DD criteria encompass diabetes’ effects on interpersonal relationships. DD can directly impact a young patient’s parents as well [30]. A potential consideration for young people with diabulimia and DD may be the integration of family therapy into the recovery plan. Family therapy can be key to recovery, as poor family dynamics can exacerbate the patient’s condition [3]. Other useful therapy modalities for reducing DD include mindful self-compassion interventions and mindfulness-based cognitive therapy [31].

People with T1D are at increased risk of developing eating disorders [25], making it worthwhile to screen every patient with T1D for DD. The greatest potential for enhanced knowledge of DD lies in its ability to prevent disordered eating behaviors in patients with T1D. If feelings of frustration with diabetes management can lead to restriction of food or insulin, then screening for DD could uncover distress before patients begin to act on it.

### Limitations and future directions

While the studies surveyed in this review contained valuable insight from patients with diabulimia and eating disorders in the setting of T1D, none of the studies specifically sought to quantify their symptoms using a diabetes distress assessment tool. Although many of the patients reported symptoms associated with DD, it is impossible to know how many of them would have been diagnosed with DD, nor can we ascertain severity, had such an assessment been administered. Thus, while we have estimates of the prevalence of diabulimia and DD within the T1D population, the prevalence and severity of DD within the diabulimia population remain unknown. Future research should continue to investigate the links between DD and diabulimia; a study administering DDS assessments to patients with diabulimia would be especially helpful. While this paper does not demonstrate a causative relationship between DD and diabulimia, it brings sufficient evidence for a likely association between the two. Because of the potentially lethal complications that arise from diabulimia, DD screening in every patient with diabetes could one day be a vital tool for prevention and early intervention.

## Conclusion

Due to the inter-specialty overlap inherent in diabulimia and DD, healthcare professionals in all disciplines must become comfortable in screening for, diagnosing and treating diabulimia and DD. Providers will need to anticipate the psychological effects of day-to-day diabetes self-management and be aware that feelings of distress could potentially lead to diabulimia or other disordered eating behaviors. Overall, this paper demonstrates the role of DD in patients who develop diabulimia and offers considerations for clinical practice and future research.

## Data Availability

All data was sourced from studies published on PubMed.
